# Association Between Dynamic Trends of Functional Disability and Poverty Among People Aged 45 and Over

**DOI:** 10.3389/fpubh.2021.742385

**Published:** 2022-01-17

**Authors:** Hui Liao, Chaoyang Yan, Ying Ma, Jing Wang

**Affiliations:** ^1^Department of Health Management, School of Medicine and Health Management, Tongji Medical College, Huazhong University of Science and Technology, Wuhan, China; ^2^The Key Research Institute of Humanities and Social Science of Hubei Province, Huazhong University of Science and Technology, Wuhan, China; ^3^Institute for Poverty Reduction and Development, Huazhong University of Science and Technology, Wuhan, China

**Keywords:** functional disability, dynamic trends, economic conditions, poverty, catastrophic health expenditure

## Abstract

**Background:**

The disability problem has become prominent with the acceleration of the global aging process. Individual disability is associated with economic conditions and contributes to family poverty. As disability will change over a long period of time and may even show distinct dynamic trends, we aimed to focus on activities of daily living (ADL) and classify functional disability trends. Moreover, we aimed to highlight and analyze the association between functional disability trends and economic conditions and explore the influencing factors.

**Materials and Methods:**

A total of 11,222 individuals who were 45 years old or older were included in four surveys conducted by the China Health and Retirement Longitudinal Study in 2011, 2013, 2015, and 2018. Samples were analyzed after excluding those with missing key variables. The latent class growth model was used to classify the ADL trends. Two binary logistic regressions were established to observe the association between the ADL trends and follow-up economic conditions or catastrophic health expenditure trends.

**Results:**

ADL trends of older adults were classified into improving (25.4%), stabilizing (57.0%), and weakening ADL (17.6%). ADL trend was associated with follow-up poverty (*p* = 0.002) and catastrophic health expenditure trends (*p* < 0.001). Compared with the improving ADL trend, the stabilizing ADL may have a negative influence on individuals' economic conditions (OR = 1.175, 95%CI = 1.060–1.303). However, a stabilizing ADL trend was less likely to bring about catastrophic health expenditures (OR = 0.746, 95%CI = 0.678–0.820) compared with an improving ADL trend.

**Conclusion:**

The improvement of functional disability would make the medical expense burden heavier but would still be beneficial for the prevention of poverty. A significant association was found between socioeconomic factors and poverty. Preventing the older adults from developing disability and illness, improving the compensation level of medical insurance, and optimizing the long-term care insurance and the primary healthcare system can potentially contribute to the prevention of poverty. Meanwhile, focusing on people who are poor at early stages, women, middle-aged, low-educated, and in rural areas is important.

## Introduction

Disability refers to a state of being unable to take care of oneself completely in daily life due to aging, weakness, illness, and physical or mental dysfunction, and other reasons for a long period of time (usually more than 6 months) (1). The World Health Organization (WHO) defines disability even more broadly; it covers impairments, activity limitations, and participation restrictions. A disabled person is someone with problems in body function or structure, as well as someone who experiences difficulties in carrying out a task, action, or problems associated with life situations. Although disability can have several definitions, the activities of daily living (ADL) and instrument ADL (IADL) are considered the most common (2). Meanwhile, a disability may change over time with aging, particularly long-term disabilities. Disability can accurately reflect and evaluate the individual's health status over a long period of time.

The disability problem has become prominent with the acceleration of the global aging process. In the United States, up to 61% of community-dwelling older adults had at least one functional difficulty in 2016 (3). In Europe, the population with disability probably accounts for 17% of persons between the ages of 16 and 64 years. Among them, 12.3% had moderate limitations on their abilities and 4.7% were severely disabled in 2018 (4). In South Korea, the proportion of people with disabilities who were 65 years old and older had increased dramatically from 30.3% in 2000 to 43.3% in 2014; this rate is over three times higher than the growth rate of the overall aging population (5). According to the National Bureau of Statistics of China, the number of disabled elderly exceeded 44 million, which accounted for 19.1% of the total elderly population (6). Some studies have predicted that the number of disabled elderly in China will reach 76.11 million in 2030 and 120 million in 2050 (7).

Individual disability is associated with economic conditions. It influences the earnings and medical expenses and affects the incidence of poverty. Some studies found that poverty is typically accompanied by disability. Rosano et al. found that the number of households under the poverty line with a disabled family member reached 68% in Italy (8). Regarding the effect of disability on the entire family's economic conditions, an existing study reveals that poverty levels in households with disabled members were higher than those of the general population in the UK (9). For the individual, Hyde and Meyer et al. confirmed that the earnings of people with a chronic or severe disability usually decline (10, 11). The average earnings of people who have been disabled for 10 years dropped by 77% (10). Dai et al. further suggested that the health expenditures of older adults with functional limitations are higher compared with those with chronic diseases alone (12). Some studies found that older adults with disabilities are the most vulnerable to poverty and ill health. For instance, compared with older adults without disability, the rate of disabled older adults whose earnings are lower than the minimum cost of living is higher (67.3%) (13), and the prevalence of diabetes is higher among individuals living with either a physical or intellectual disability (14).

Although previous studies have investigated the transitions of disability over time and the association between disability and economic conditions, few studies have focused on distinct dynamic trends of disability condition and economic consequence, especially poverty among older adults. Individual disability conditions will change over time and may be related to some other factors, which have been confirmed in some studies. For example, Rector et al. found that faster accumulation of chronic conditions and a steeper decline in activity were associated with a greater increase in functional limitations over time (15). Moreover, recent analyses have indicated that apart from enduring disability, individuals also experience transitory disability over time, and the specific strategies for addressing these types of disability may vary (16, 17). The quality of life and welfare of people with different development trends of disability would decline in various degrees, owing to unequal financial loss, which is aggravated by the declining state of ability or unequal medical expenses caused by the demand for healthcare service. Accordingly, this study aimed to fill the knowledge gap by determining the effect of different disability trends on economic conditions, which include poverty and medical expenses.

Some confounding factors, such as demographic and socioeconomic factors, may affect the disability conditions. Socioeconomic status disparities exist in late-life disability in European countries, such as Denmark (18), England (19), Italy, and the Netherlands (20); Asian countries including China (21) and Japan (22); and the U.S. (23). This finding indicated that disability is related to socioeconomic factors. Among the specific demographic details, factors such as age, gender, and education are strongly correlated with disability. Sugisawa concluded that income disparities related to late-life disability decreased with advancing age (24), suggesting that age affects the association between income and disability. Murtagh and Wray et al. stated that women have a higher prevalence of disability in IADL and ADL than men (25, 26). Mackenbach et al. showed that poorly educated or deprived people are at a higher risk of disability than well-educated or better-off people (27). Thus, studying whether confounding factors will affect the association between the dynamic changes in disability and economic conditions is necessary to control these confounding factors. Results would help with the development of effective measures to prevent health-related poverty from a multidimensional perspective.

Considering that the individuals' disability may change and show distinct trends over a long period of time, and the association between the dynamic changes in disability over time and economic conditions and also the influencing factors is unclear, this study aimed to focus on ADL and achieve the following goals: (1) to classify the types of functional disability trends in older adults; (2) to analyze the association between functional disability trends and economic conditions of older adults; and (3) to explore the factors affecting the association between functional disability trends and economic conditions in a sample of older adults aged 45 years old and older in China.

This study will help support the dynamic control of disability and further prevent poverty due to illness. The research on the association between functional disability trends and poverty and its influencing factors in China, the largest developing country in the world, can be meaningful in revealing the relationship between healthcare improvement and economic development, especially for developing or underdeveloped areas of the world.

## Materials and Methods

### Data Source and Sample Selection

The data used in this study were panel data extracted from the China Health and Retirement Longitudinal Study (CHARLS) in 2011, 2013, 2015, and 2018. CHARLS is a longitudinal survey that collects the health and aging status of middle-aged and elderly population aged 45 years old and older in China. The national baseline survey of CHALRS was conducted since 2011, which covers 150 counties and 450 villages. A follow-up survey was conducted every 2–3 years. CHARLS has fulfilled regular surveys in 2011, 2013, 2015, and 2018. Moreover, the questionnaire of the CHARLS regular survey contains information regarding basic demographics, family structure, financial support, health status, health care, employment, household economy (income, consumption, and wealth), and the basic situation of the community.

**Figure 1:**
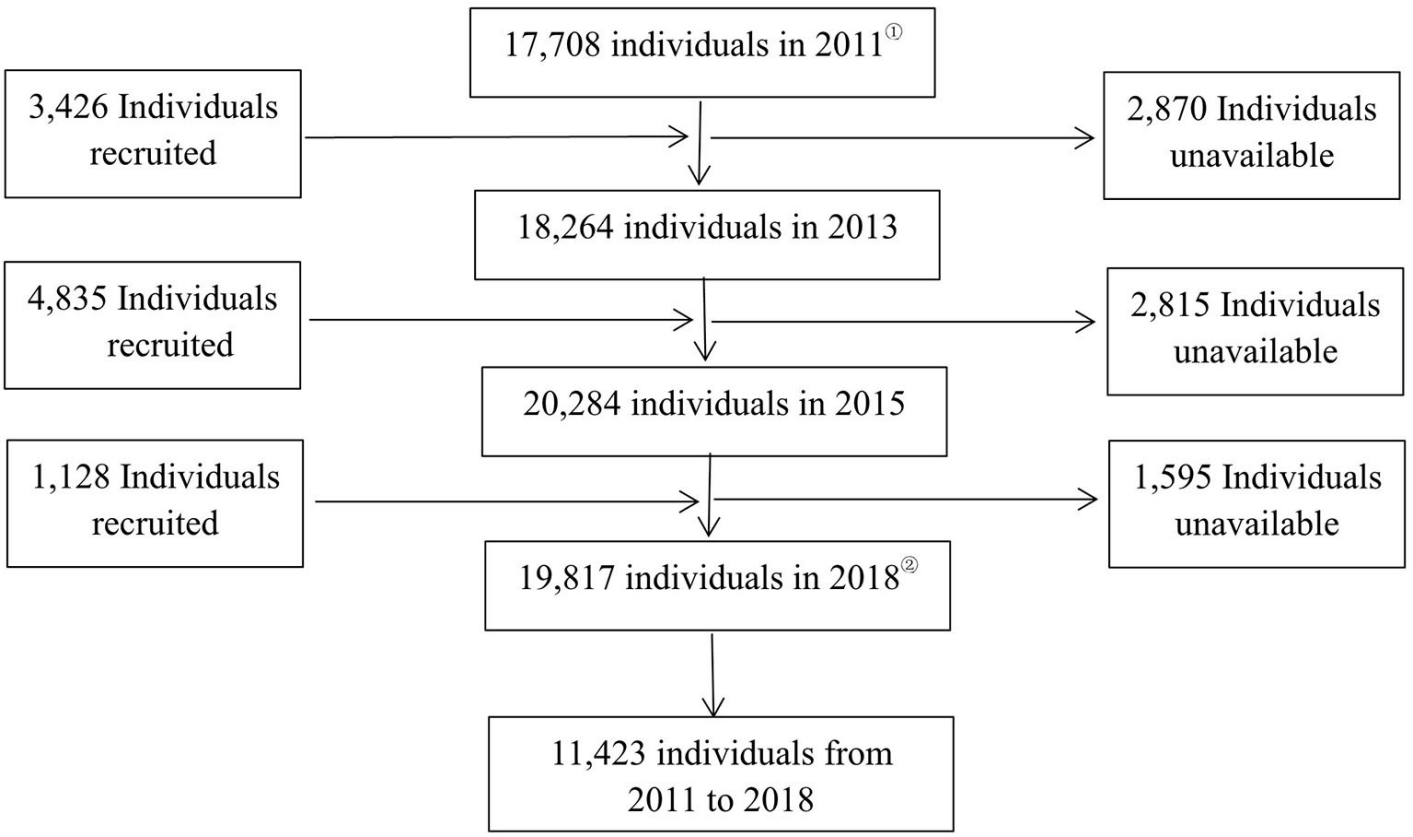
Sampling flowchart. ① 2011 is the T(0), ② 2018 is the T(n).

The regular survey adopts multistage sampling, and the probability proportional to size (PPS) sampling method is adopted in the sampling stage of counties/districts and villages. First, 150 districts and counties were randomly selected from 30 provincial administrative units across the country (excluding Tibet, Taiwan, Hong Kong, and Macao). Then, three villages or communities were randomly selected from each of the 150 districts and counties. Finally, 450 villages/communities were obtained. The samples in the 2011, 2013, 2015, and 2018 surveys were 17,708 individuals from 10,251 households, 18,264 from 10,629 households, 20,284 from 11,797 households, and 19,817 from 12,400 households after excluding samples that were lost to follow-up. A total of 11,423 individual panel data were covered by the four surveys. In this study, the baseline year 2011 was T(0) and the follow-up year 2018 was T(n). A total of 11,222 individuals who were 45 years or older and covered by the four surveys were analyzed after excluding 201 samples with missing key variables regarding disability (Figure 1).

### Variables

#### Dependent Variable

The main dependent variables were the T(n) poverty and the trend in catastrophic health expenditure (CHE). Poverty was judged by the total household expenditure and number of people living in that household. The direct measurement index of poverty is per capita annual net income. China's national poverty line is the 2011 per capita net income of 2,300 CNY. After consumer price index adjustment, the national poverty line was converted to 2,649.6 CNY in 2018. According to the US$ average exchange rate in 2018 (1 USD = 6.62 CNY), this was equivalent to 400.24 USD. This study substituted the expenditure for income; thus, an annual household expenditure per capita of below 400.24 USD in 2018 was defined as poverty.

Catastrophic health expenditure is defined as household out-of-pocket (OOP) medical expenses that exceed certain fractions of the total household expenditure or non-food expenditure. Generally, three thresholds are widely used to define CHE, as follows: (1) OOP medical expenses that exceed 10% of the total expenditure (28, 29); (2) OOP medical expenses that exceed 30% of the total expenditure (30); and (3) OOP medical expenses that exceed 40% of the non-food expenditure (31, 32). This study selected the threshold value of 30% to define CHE, and the data came from the household living expenditure of CHARLS in 2011, 2013, 2015, and 2018. If the OOP medical expenses in 1 year accounted for <30% of the total household expenditure, then the household did not have CHE during the year. If the proportion of OOP medical expenses exceeded 30%, then the household had CHE in that year. From this criterion, the study obtained the CHE conditions in the households of the respondents in 2011, 2013, 2015, and 2018. Furthermore, the CHE trends were classified by latent class growth model and regarded as another dependent variable. Specific classification of CHE trends will be introduced in the following statistical analysis and results sections.

The total household expenditure is among the criteria for measuring poverty and can reflect the general economic conditions of a household. In the retrospective survey, it was more difficult to measure the income accurately due to its complex sources and uncertainty, especially for most of the village residents. Hence, this study adopted accurate and objective expenditure indicators. Some studies have used total household expenditure to measure the poverty of a household with CHARLS or other surveys (33–35). Therefore, this study used the total household expenditure to construct poverty variables for measuring economic conditions.

#### Independent Variables

In this study, ADL was used to assess functional disability. ADL trend and T(0) poverty were assumed as the independent variable. The data about ADL trend came from the functional limitations and helpers of CHARLS 2011, 2013, 2015, and 2018 data. The data of T(0) poverty came from the parent childrearing, sibling information, and household expenditures of CHARLS 2011.

In the ADL, the CHARLS asked about six items, namely, dressing, bathing, eating, getting into or out of bed, using the toilet, and controlling urination and defecation. For each item, four options were designed, as follows: (1) No, I do not have any difficulty; (2) I have difficulty but can still do it; (3) Yes, I have difficulty and need help; and (4) I cannot do it. To explore the change in disability condition, this study categorized ADL as non-disability and disability (partial and full). In accordance with the methods of Meinow et al. (36) and Gomi et al. (37), this study designated the score of 0 for having no difficulty; 1 for having difficulty but can still do it; and 2 for having difficulty, needing help, and cannot do it. The total scores corresponding to the six ADL items were between 0 and 12. An ADL total score of 0 was considered to have no disability, and an ADL total score of between 1 and 11 was considered to have partial disability. Conversely, a score of 12 was considered to have full disability. Finally, the distinct ADL trends were classified by latent class growth model. Specific classification of ADL trends will be introduced in the following statistical analysis and results sections.

The T(0) poverty was judged by the total household expenditure and the number of people living in this household. The direct measurement index of poverty was per capita annual net income. China's national poverty line in 2011 is the per capita net income of 2,300 CNY. According to the US$ average exchange rate in 2011 (1 USD = 6.46 CNY), this amount was equivalent to 356.04 USD. This study substituted expenditure for income. Thus, an annual household expenditure per capita of below 356.04 USD in 2011 was defined as poverty.

#### Control Variables

Control variables in this study came from the demographic background part of CHARLS in 2011 and 2018, which included gender, age, education, marital status, and residence. Table 1 shows the detailed definitions and coding of each control variable.

**Table 1:** Code and question description of variables.

**Variables**	**Code**	**Question description**
T(0)/T(n) gender	0 = male, 1 = female	Interviewer recorded R's gender
T(0)/T(n) age	0 = 45–60, 1 ≥ 60	What is your actual date of birth
T(0)/T(n) education	0 = no formal education, 1 = elementary school and below, 2 = middle school, 3 = high school and above	Has your highest level of education changed from the last wave? If so, what is the highest level of education you have attained now (not including adult education)?
T(0)/T(n) marital status	0 = married, 1 = unmarried	What is your marital status?
T(0)/T(n) residence	0 = urban community, 1 = rural village	Was the type of address village or city/town?
T(0)/T(n) ADL	0 = no disability, 1 = partial disability, 2 = full disability	
ADL trend	0 = improving ADL, 1 = stabilizing ADL, 2 = weakening ADL	
T(0) poverty	0 = no, 1 = yes	
T(n) poverty	0 = no, 1 = yes	
CHE trend	0 = the probability of CHE is less, 1 = the probability of CHE is greater	

### Statistical Analysis

First, the study used the latent class growth model (LCGM) to classify the changing trend of the respondents' ADL and CHE. The LCGM operates from the assumption that there are meaningful unobserved subpopulations within the larger sample, each with a distinct longitudinal trajectory (38). Hence, it is a method that can be applied to longitudinal data and latent class variables to describe the development feature of samples in a certain period and classify the development feature. We considered small values of Akaike Information Criterion (AIC) and Bayesian Information Criterion (BIC), and adjusted the Bayesian Information Criterion (aBIC) to select the number of latent classes (39).

Then, the study used the Chi-square test to describe the distribution of distinct ADL trends in the T(0) economic conditions and to observe whether different economic conditions had significant differences in various ADL trends. The T(0) economic conditions were measured by poverty.

Next, the study used a binary logistic regression to analyze the association between the ADL trend and T(n) economic conditions and to observe whether the changes in ADL would affect T(n) economic conditions. If an influence existed, then the direction of change was observed in this association. The T(n) economic conditions were measured by poverty.

Finally, a binary logistic regression was used to analyze the association between the ADL trend and CHE trend, including the confounding factors of T(0) poverty and socioeconomic status. Whether ADL trend and confounding factors affected the CHE trend was examined. If there were effects, then the direction of change in the association between them was explored. Thus, the influencing factors in the association between ADL trend and economic conditions were explored.

The LCGM was applied using Mplus 8. The Chi-square analysis and logistic regression were executed using SPSS12 with the statistical significance at *p* < 0.05.

### Ethics Statement

The CHARLS study data are publicly available and open to researchers worldwide. Ethics approval for data collection in CHARLS was obtained from the Biomedical Ethics Review Committee of Peking University (IRB00001052–11015). All respondents were required to sign their consent under conditions of privacy after clarifying that the decision to participate in this research was entirely voluntary.

## Results

Table 2 shows the model fitting results of the latent class growth model for ADL and CHE. We classified the trends of ADL into three classes and the trends of CHE into two classes while considering the criteria, which included small values of BIC, aBIC, and AIC, along with an entropy score close to 1 (40). According to the estimated posterior probabilities, the proportions of ADL trend divided into three classes were 17.6, 57.0, and 25.4%, and the two classes of CHE trends were 59.3 and 40.7%. Classes that are <10% of the total sample may represent accidental discovery and were unsuitable for inclusion in the study (40, 41). Thus, three classes of ADL trends and two classes of CHE trends were the most acceptable. The two classes of CHE trends were composed of different population incidences of CHE in the four waves of the survey. Thus, the class with the population incidence of CHE in slow growth was defined as “the probability of CHE is less,” but one that was increasing rapidly was regarded as “the probability of CHE is greater”.

**Table 2:** Model fit indices for longitudinal latent class analysis on ADL and catastrophic health expenditure over time.

**Class**	**AIC**	**BIC**	**aBIC**	**Entropy**
**Panel ADL**				
2 (n1 = 25.4%, n2 = 74.6%)	24907.584	24995.491	24957.357	1.000
3 (n1 = 17.6%, n2 = 57.0%, n3 = 25.4%)	23004.984	23114.869	23067.201	0.990
4 (n1 = 0.0%, n2 = 57.0%, n3 = 25.4%, n4 = 17.6%)	23010.984	23142.846	23085.644	0.992
**Panel catastrophic health expenditure**				
2 (n1 = 59.3%, n2 = 40.7%)	4697.533	4785.441	4747.306	0.985
3 (n1 = 40.7%, n2 = 59.3%, n3 = 0.0%)	4703.533	4813.418	4765.749	0.991

**Figure 2:**
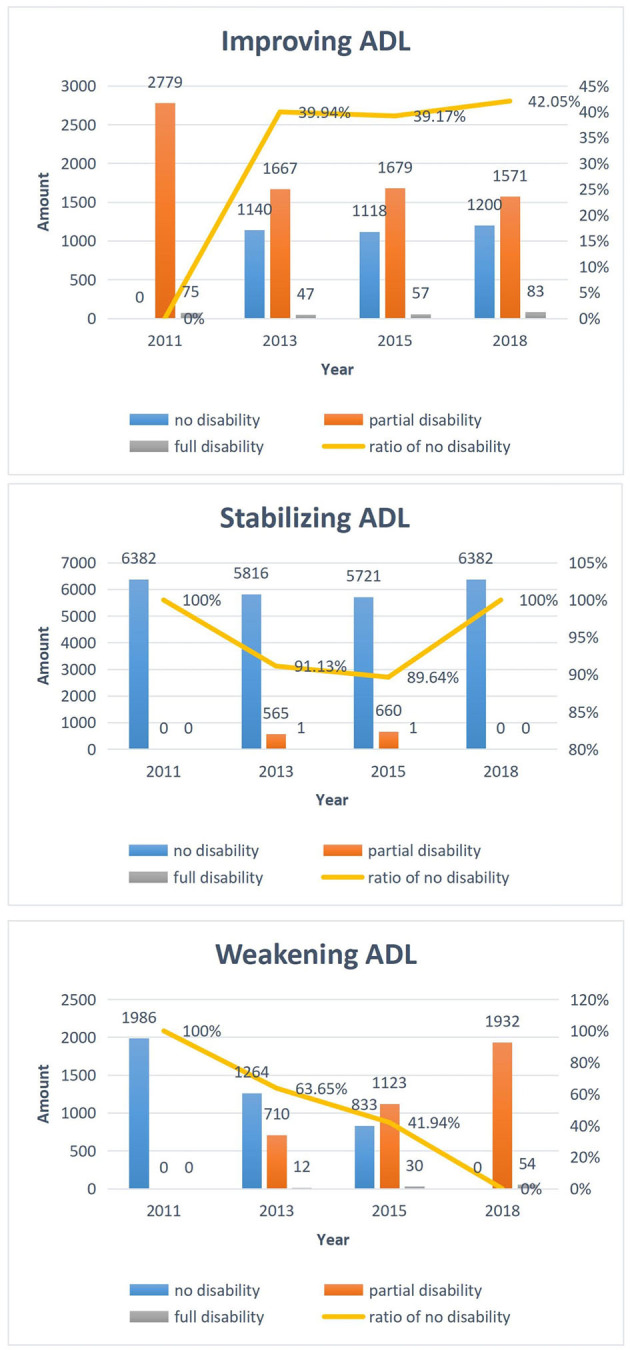
Three classes of ADL trends and their composition of disability condition in four waves.

Figure 2 shows the three classes of ADL trends and the composition of different disability condition in four waves in each class. The class with a proportion of 25.4% showed an overall trend of improvement in ADL because the number of individuals with no disability tended to increase. However, those with partial disability tended to decrease. The ratios of no disability, partial disability, and full disability were 0.0, 97.4, and 2.6% (2011); 39.9, 58.5, and 1.6% (2013); 39.2, 58.8, and 2.0% (2015); and 42.0, 55.0, and 3.0% (2018), respectively. The class with a proportion of 57.0% showed an overall trend of stabilizing ADL, in which the ratios of no disability, partial disability, and full disability were 100.0, 0.0, and 0.0% (2011); 91.1, 8.9, and 0.0% (2013); 89.6, 10.3, and 0.1% (2015); and 100.0, 0.0, and 0.0% (2018), respectively. Moreover, an overall trend of weakening ADL was observed in the class with a proportion of 17.6% because the number of individuals with no disability tended to decrease, but that with partial disability tended to increase. The ratios of no disability, partial disability, and full disability were 100.0, 0.0, and 0.0% (2011); 63.6, 35.8, and 0.6% (2013); 41.9, 56.6, and 1.5% (2015); and 0.0, 97.3, and 2.7% (2018), respectively. These results reflected that the functional disability trend of older adults would be recovering, stable, or worsening. Moreover, the functional disability conditions of most older adults tended to be stable, and the most disabled people had partial disability instead of full disability.

Table 3 shows the sample characteristics in T(0) and the ADL trend difference within subgroups of T(0) poverty or T(0) control variables. In total, the numbers of male and female participants were nearly the same, with males accounting for 42.3% and females accounting for 57.7%. Moreover, 52.5% of the individuals were 45–60 years old, and 47.5% were over 60 years old. Their average age was 61.02 ± 0.097 years (Mean ± SD). Only 9.0% of the participants had an education level of high school or above, and more participants lived in rural areas (63.0%) and were married (99.0%). The respondents who were in poverty in the T(0) period accounted for 56.0%.

**Table 3:** Sample characteristics in T(0) and the ADL trends comparison within subgroups of T(0) economic conditions or T(0) control variables.

**Item**		**Total**	**ADL trends (n/%)**				
		**(n/%)**	**Improving ADL**	**Stabilizing ADL**	**Weakening ADL**	**χ^2^**	**P**
Control variables	T(0) gender					19.471	0.000
	Male	4,747 (42.3)	1,189 (25.0)	2,797 (58.9)	761 (16.0)		
	Female	6,475 (57.7)	1,665 (25.7)	3,585 (55.4)	1,225 (18.9)		
	T(0) age					460.006	0.000
	45–60	5,889 (52.5)	1,098 (18.6)	3,899 (66.2)	892 (15.1)		
	>60	5,333 (47.5)	1,756 (32.9)	2,483 (46.6)	1,094 (20.5)		
	T(0) education					356.602	0.000
	No formal education	3,760 (33.5)	1,217 (32.4)	1,771 (47.1)	772 (20.5)		
	Elementary school and below	4,554 (40.6)	1,139 (25.0)	2,583 (56.7)	832 (18.3)		
	Middle school	1,899 (16.9)	350 (18.4)	1,294 (68.1)	255 (13.4)		
	High school and above	1,009 (9.0)	148 (14.7)	734 (72.7)	127 (12.6)		
	T(0) marital status					9.694	0.008
	Married	11,114 (99.0)	2,813 (25.3)	6,334 (57.0)	1,967 (17.7)		
	Unmarried	108 (1.0)	4 1(38.0)	48 (44.4)	19 (17.6)		
	T(0) residence					86.489	0.000
	Urban community	4,155 (37.0)	901 (21.7)	2,597 (62.5)	657 (15.8)		
	Rural village	7,067 (63.0)	1,953 (27.6)	3,785 (53.6)	1,329 (18.8)		
Independent variables	T(0) poverty					2.260	0.323
	No	4,943 (44.0)	1,261 (25.5)	2,837 (57.4)	845 (17.1)		
	Yes	6,279 (56.0)	1,593 (25.4)	3,545 (56.5)	1,141 (18.2)		
	T(0) ADL					11222.000	0.000
	No disability	8,368 (74.6)	0 (0.0)	6,382 (100.0)	1,986 (100.0)		
	Partial disability	2,779 (24.8)	2,779 (97.4)	0 (0.0)	0 (0.0)		
	Full disability	75 (0.7)	75 (2.6)	0 (0.0)	0 (0.0)		
	T(n) ADL					8079.936	0.000
	No disability	7,582 (67.6)	1,200 (42.1)	6,382 (100.0)	0 (0.0)		
	Partial disability	3,503 (31.2)	1,571 (55.0)	0 (0.0)	1,932 (97.3)		
	Full disability	137 (1.2)	83 (2.9)	0 (0.0)	54 (2.7)		

The ADL trend was significantly related to gender, age, education, marital status, and residence. The class of stabilizing ADL consisted of more people with ages in the range of 45–60 years and were indicated as men, with higher education, married, and living in an urban community. Additionally, the classes of improving ADL and weakening ADL consisted of more people with ages of over 60 years and were indicated as women, with lower education, and living in rural villages. The trend of improving ADL was greater for unmarried people, whereas that of weakening ADL was greater for married people. Moreover, according to the ADL from T(0) to T(n), the increased number of non-disability samples was distributed in the class of improving ADL, whereas the increased number of samples with partial and full disability was distributed in the class of weakening ADL. There was no change in the class of stabilizing ADL.

Table 4 shows the association between ADL trend and T(n) poverty. ADL trend was significantly related with T(n) poverty, and the ADL trend was a risk factor for its OR of > 1. Thus, those individuals whose functional disability was stable were 1.175 times more likely to fall into poverty than those with improving functional disability. This result indicated that functional disability improvement may be associated with better economic conditions.

**Table 4:** T(n) poverty models on ADL trend and control variables.

**Variables**		**OR**	**95% CI**	***P***
ADL trend (Ref improving ADL)	Stabilizing ADL	1.175	1.060–1.303	0.002
	Weakening ADL	0.988	0.867–1.126	0.859
T(0) poverty (Ref no)	Yes	3.180	2.903–3.484	0.000
T(n) gender (Ref male)	Female	1.118	1.019–1.226	0.018
T(n) age (Ref 45–60)	>60	0.889	0.802–0.986	0.026
T(n) education (Ref no formal education)	Elementary school and below	1.002	0.906–1.109	0.962
	Middle school	0.967	0.841–1.113	0.644
	High school and above	0.688	0.564–0.839	0.000
T(n) marital status (Ref married)	Unmarried	0.844	0.537–1.327	0.462
T(n) residence (Ref urban community)	Rural village	2.432	2.178–2.714	0.000
Constant		0.108		0.000

The T(n) poverty was significantly related to T(0) poverty, T(n) gender, T(n) age, T(n) education, and T(n) residence, but T(n) marital status was insignificantly related to T(n) poverty. Among the significant control variables, T(0) poverty, gender, and residence were risk factors, because their OR was > 1, whereas age and education were protective factors because their OR was <1. Thus, people whose families were poor at an early stage were 3.180 times more likely to be poor in the end. Women were 1.118 times more likely to fall into poverty than men. Those who lived in rural villages were 2.432 times more likely to fall into poverty than those who lived in urban communities. Middle-aged people were 0.889 times more likely to be poor than the elderly, and those whose highest level of education is high school and above were 0.688 times more likely to fall into poverty than those who did not receive formal education. These results indicated that the economic conditions of people who were poor at early stages, women, middle-aged, living in rural village, or with lower education would be worse.

Table 5 shows the association between ADL and CHE trends. ADL trend was significantly related with CHE trend, and the ADL trend was a protective factor because its OR was <1. Thus, individuals whose functional disability was stable were 0.746 times more likely to have CHE than those with improving functional disability. This result showed that improving functional disability may be associated with greater risk of CHE.

**Table 5:** Catastrophic health expenditure trend models on ADL trend and control variables.

**Variables**		**OR**	**95% CI**	***P***
ADL trend (Ref improving ADL)	Stabilizing ADL	0.746	0.678–0.820	0.000
	Weakening ADL	0.950	0.842–1.072	0.409
T(0) poverty (Ref no)	Yes	0.558	0.516–0.604	0.000
T(0) gender (Ref male)	Female	0.886	0.815–0.963	0.004
T(0) age (Ref 45–60)	>60	1.255	1.157–1.361	0.000
T(0) education (Ref no formal education)	Elementary school and below	0.881	0.802–0.969	0.009
	Middle school	0.766	0.676–0.867	0.000
	High school and above	0.696	0.595–0.813	0.000
T(0) marital status (Ref married)	Unmarried	0.654	0.445–0.963	0.032
T(0) residence (Ref urban community)	Rural village	0.962	0.885–1.046	0.366
Constant		2.730		0.000

Catastrophic health expenditure trend was significantly related to T(0) poverty, T(0) gender, T(0) age, T(0) education, and T(0) marital status. Among the significant control variables, only age was a risk factor because its OR was > 1. T(0) poverty, gender, education, and marital status were protective factors because their ORs were <1. Thus, people aged over 60 years old were 1.255 times more likely to have CHE. People whose families were poor at early stage were 0.558 times more likely to have CHE. Women were 0.886 times more likely to have CHE than men. Unmarried people were 0.654 times more likely to incur CHE than married people. Compared with individuals who did not receive formal education, people whose educational attainment was elementary and below were 0.881 times more likely to incur CHE. Those who received middle school education were 0.766 times more likely to incur CHE. Those who received high school education and above were 0.696 times more likely to incur CHE. These results indicated that the health and economic risk for the elderly would be greater, whereas that for those who are poor at early stage, female, unmarried, and higher-educated would below.

## Discussion

The changing trend of functional disability over time had three classes, which showed that the functional disability condition of older adults would improve, remain stable, or worsen. The disability improvement may reduce the economic loss of the household gradually and prevent household poverty. However, the health and economic risk would still increase during the improvement process compared with the condition of non-disability. Moreover, the poverty risk of people who were poor at early stage, female, middle-aged people, low-educated people, and rural residents was greater. Such results meant that the heterogeneous dynamic trends of functional disability related to economic conditions could be affected by some socioeconomic factors. The possible reasons for these results could originate from the changes in individual physical function and health, large OOP medical expenses, and the capability to resist health risks.

On the one hand, the changes in individual physical function and health could affect economic conditions. First, the individuals' work ability tends to be weaker when their physical activity and health conditions deteriorate with age. Accordingly, many older adults, especially middle-aged people with disability, may lose work productivity and even lose their jobs. On the contrary, middle-aged people could engage in labor and production after the recovery of physical function to prevent themselves from losing a large portion of their income and falling into poverty. This finding is similar to that of Periyakoil and Polvinen et al. The former found that disability becomes more severe with age and that it inevitably continues to advance into end-stage diagnoses, such as fragility (42). The latter further discovered that long periods of disability may contingently lead to a high risk of economic difficulties for disabled retirees and their family (43). Second, older adults with disability during long-term treatment and rehabilitation may also suffer from a series of complications and chronic diseases, thereby increasing the medical expenses, including drug, outpatient, and hospitalization fees. Rivera-Almaraz et al. demonstrated that the rising prevalence of chronic diseases among the disabled elderly increased their medical expenses (44). Reichard et al. and Johan et al. found that disability had a significantly positive effect on the utilization and scope of medical services and medical expenses (45, 46). Thus, the changes in physical function and health supported our findings that improving disability would prevent poverty, but possibly contributes to the risk of CHE.

Large OOP medical expenses could push individuals or families into poverty. In our study, the probability of CHE was greater in the population with improving disability, which indicated that OOP healthcare payments of families were still high. First, individual medical expenses may be inadequately shared, owing to the low reimbursement ceilings of medical insurance. Statistics showed that in 2018, personal hygiene expenditure in China's total health expenditure accounted for ~40%, which was nearly twice the proportion of personal hygiene expenditure in the European Union (EU) countries, according to the statistical caliber of EU countries (47). Thus, the personal medical burden remains heavy. Second, China's long-term care (LTC) system started late and developed relatively slowly compared with the systems in European and American countries (48). Thus, high care costs were incurred due to the urgent need for LTC of disabled older adults. For example, the LTC insurance reduces the demand for hospitalization services and medical expenses by subsidizing home care and institutional care, as confirmed by Kim and Wang et al. (49, 50). However, the provision of LTC services, such as policy formulation, operational models, and fundraising, lagged far behind in China, and the piloting of the current LTC insurance system continued with limited coverage, thereby affecting insignificant cost reduction (51). Third, Chinese community-based healthcare delivery and primary care have limited medical resources, weak quality assurance, and low trust from residents. Older adults have poor access to timely and effective health services and may need to make larger healthcare payments and suffer from the burden of expense due to frequent treatment and care in tertiary hospitals (52). Compared with Australia whose focus is on primary care, it has been demonstrated that improved access to primary health care responds to the needs of people and is both cost-effective and associated with better health outcomes (53).

The lack of capability to resist health and economic risks possibly encourages the poor economic condition of people who are poor at early stage, women, middle-aged, low-educated, and in rural villages. People who were poor at an early stage may always lack adequate resources to rise from poverty, and women may possess relatively few assets and have a weak position in the labor, credit, and insurance market (54, 55). Once middle-aged people suffer from health shocks such as disability, they may be unable to work. In that case, the middle-aged people would suffer from a greater income loss and would lack financial advantages, such as pension insurance and old-age allowance, compared with the elderly. People with low education are mostly engaged in high-risk and low-income occupations. They also lack correct health care awareness, which easily worsens their health status and leads to more medical expenses. This finding is consistent with that obtained in the study conducted by Kuh et al. who assumed that low educational level may increase the risk of hazardous occupations, low income, and more chronic diseases based on the life course model (56). Restricted by primary care resources and quality, it is difficult for residents living in rural and remote areas to effectively improve their own physical function and health; thus, they are at a risk of poverty.

This study has several limitations. First, this study only used self-reported information, which is susceptible to recall bias and can be difficult to interpret due to variations in individual awareness and judgement of objective situation (57). In particular, when judging the functional disability, this study did not consider the medical diagnosis over the years, which also caused a major threat to internal validity of the reported findings. Moreover, some questionnaires were possibly completed by proxy, which may lead to inaccurate information. However, any retrospective research has recall bias and some inaccurate information. Thus, our study cannot avoid such shortcomings. Second, there may be some issues with poverty construction by using expenditures. People with high income could have saved a large proportion of their income rather than spending it, but these people were still categorized as being in poverty. However, because these issues are difficult to avoid, and limitations with income exist, our study adopted expenditure indicators. Third, a latent class growth analysis of the 4-year household expenditures for classifying different trends of poverty development was not attempted by using panel data. Based on the study of Park et al. who investigated the influence of the degree of disability on poverty dynamics (58), poverty dynamics are classified into four development trends, as follows: sustained poverty, sustained non-poverty, poverty exit, and poverty entry. Our study only focused on the early and late economic conditions. Thus, considering poverty dynamics will be valuable in the follow-up research.

## Conclusion

Our study found that the improvement of functional disability would make the medical expenses burden heavier, but it may be beneficial for preventing poverty. Some socioeconomic factors promote the influence of disability trends on poverty. It is urgent to promote early intervention because the possible reasons for poverty could be the change in the individual's physical function, large OOP medical expenses, and the lack of capability to resist health and economic risks. First, many measures should be implemented to reduce the individual financial burden of patients and to prevent older adults from rapidly developing disability and illness. Second, the study highlights the need to improve the compensation level of medical insurance. In particular, the LTC insurance system and primary healthcare system need to ensure adequate sharing of medical costs and sufficient and accessible medical service. Finally, we could provide policy support to individuals who are poor at an early stage, women, middle-aged, low-educated, and living in rural areas, thereby strengthening their capability to resist health risks. These tasks are not simple. Nevertheless, these tasks can be accomplished if all interests work together in an integrated and committed effort to achieve a common goal.

## Data Availability Statement

Publicly available datasets were analyzed in this study. This data can be found at: http://charls.pku.edu.cn/pages/data/111/zh-cn.html. And the names of the repository/repositories and accession number(s) can be found at this link.

## Ethics Statement

Written informed consent was obtained from the individual(s) for the publication of any potentially identifiable images or data included in this article.

## Author Contributions

HL and JW contributed to the conception and design of the study. HL, CY, and YM conducted the data reduction and analyses. HL wrote the manuscript. JW guided the whole process and reviewed the manuscript. All authors read and approved the manuscript before submission.

## Funding

This research was supported by: (1) Research on dynamic optimization of coping strategies on health poverty risk for rural older households, funded by National Natural Science Foundation of China (Grant Number 72074086); (2) Research on multidimension risk identification of health poverty vulnerability of the elderly in rural areas and targeted poverty alleviation strategy, funded by National Natural Science Foundation of China (Grant Number 71673093). The funding bodies played no role in the design of the study and collection, analysis, and interpretation of data and in writing the manuscript.

## Conflict of Interest

The authors declare that the research was conducted in the absence of any commercial or financial relationships that could be construed as a potential conflict of interest.

## Publisher's Note

All claims expressed in this article are solely those of the authors and do not necessarily represent those of their affiliated organizations, or those of the publisher, the editors and the reviewers. Any product that may be evaluated in this article, or claim that may be made by its manufacturer, is not guaranteed or endorsed by the publisher.
